# Histological validation of carotid plaque characterization by in-vivo T2 mapping in patients with recent cerebrovascular events: preliminary results

**DOI:** 10.1186/1532-429X-16-S1-P173

**Published:** 2014-01-16

**Authors:** Luca Biasiolli, Joshua T Chai, Linqing Li, Ashok Handa, Peter Jezzard, Robin Choudhury, Matthew D Robson

**Affiliations:** 1 Radcliffe Department of Medicine, University of Oxford, Oxford, UK; 2 Nuffield Department of Clinical Neurosciences, University of Oxford, Oxford, UK; 3 Nuffield Department of Surgical Sciences, University of Oxford, Oxford, UK

## Background

Multicontrast CMR is the conventional method for in-vivo characterization of carotid plaques. However, its non-quantitative nature and the need for extensive post-acquisition interpretation limit its clinical application. We have recently proposed using in-vivo quantitative T2 mapping for atherosclerotic plaque characterization [Biasiolli et al. JCMR 2013, 15:69]. T2 maps have the advantage of providing an absolute physical measure of plaque components that can be standardized among different MR systems and widely adopted in multi-centre studies. This pilot study investigates the agreement between T2 mapping and histology using AHA plaque type classification.

## Methods

CMR: 19 symptomatic patients scheduled for endarterectomy (14 males, 73 ± 11 years, range 54-89 years, IRB approved, written consent obtained) were imaged at 3T (Siemens Verio) with a pair of 2-channel phased-array carotid coils (Machnet). In addition to the conventional multicontrast CMR protocol, a novel black-blood multi-slice DANTE-prepared [Li et al. MRM 2012, 68:1423-1438] multiecho-spin-echo sequence was used to acquire 5 slices with 14 echoes (TR = 2000 ms, TE = 9-127 ms) in <4 minutes (partial Fourier = 5/8, FOV = 128 × 128, matrix size = 384 × 384, slice thickness = 2 mm, slice gap = 2 mm). DANTE parameters: flip angle = 8°, number of pulses = 120, time duration between pulses = 0.5 ms, Gz = 18 mT/m and gradient duration = 0.4 ms. T2 maps were generated by mono-exponential fitting using non-linear least-squares regression. A reviewer (L.B.) classified plaque types following the CMR-modified AHA scheme using T2 maps + TOF only. Histology: Carotid plaques were freshly obtained at the time of endarterectomy, divided at the level of maximal stenosis into two 4 mm-segments and processed for formalin-fixed paraffin-embedded (FFPE) sections and cryosections, respectively. FFPE sections were stained for H&E, Masson's trichrome, while cryosections were used for Oil-Red-O/adipophilin (foam cells marker) staining to visualize lipid. AHA type of plaque histology was determined by a reviewer (J.T.C.) blinded to the T2 map results. CMR-histology slice location matching was performed for each 4 mm segments using the carotid bifurcation as the common anatomical landmark and cross-sectional T1-weighted images of the vessel wall.

## Results

**Figure 1:**
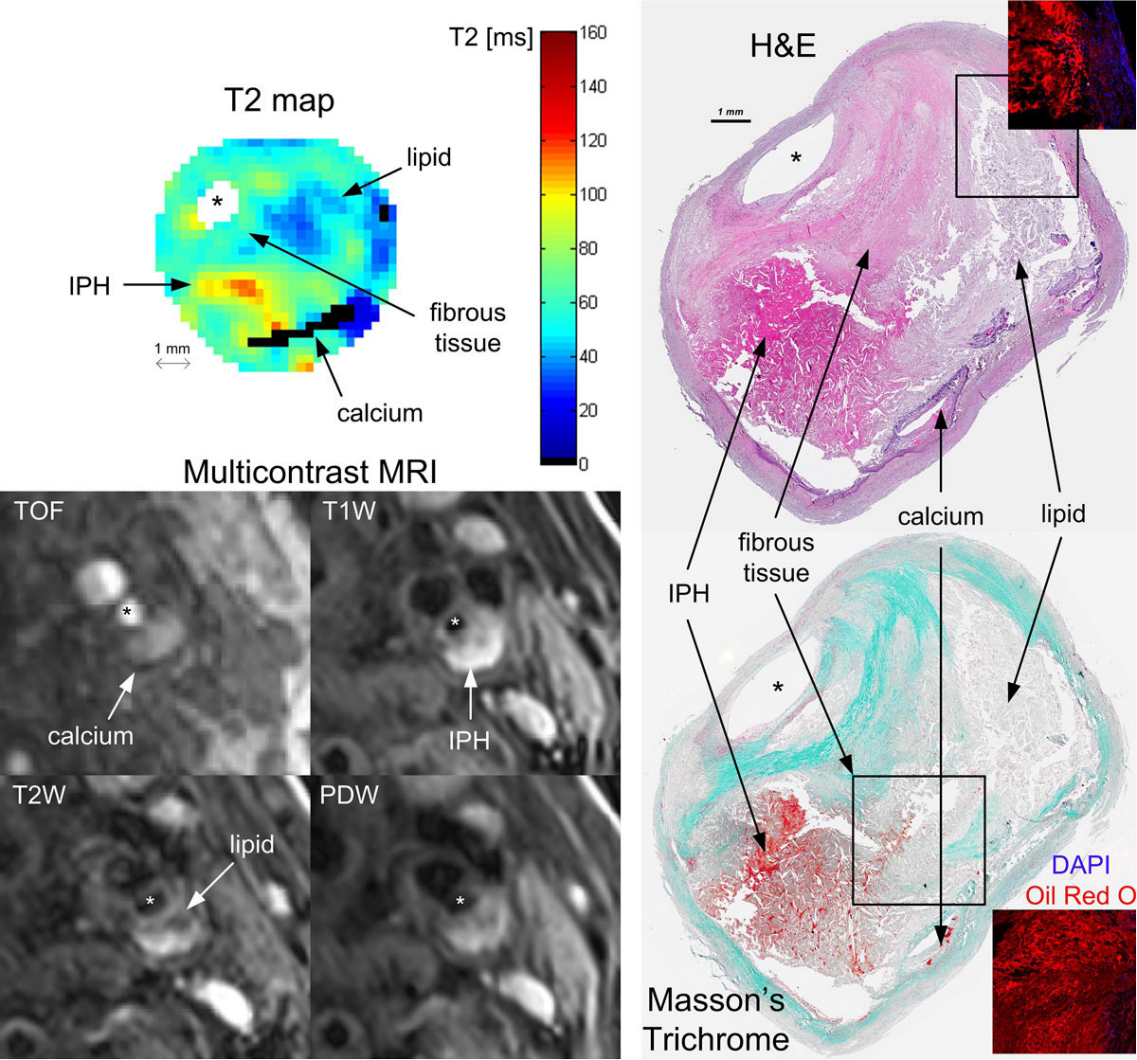
**T2 map, multicontrast MRI and histology of a type VI plaque with a large lipid-rich necrotic core (LRNC) separated from the lumen by a thick fibrous cap, clear signs of recent intraplaque-haemorrhage (IPH) and calcification**.

6 of the 19 patients scheduled for endarterectomy were excluded due to severe patient motion artefacts on CMR. The table 1 presents the AHA type classification of the remaining 13 plaques using T2 maps + TOF vs. histology. The 2 cases of misclassification were due to the difficulty of staging intraplaque haemorrhage (IPH) accurately. T2 maps were able to differentiate lipid-rich necrotic core, fibrous tissue, calcification and recent IPH, as illustrated in the Figure 1 for a type VI plaque.

**Table 1:** AHA plaque type classification by CMR (T2 maps +TOF) vs. histology

Histology	CMR (T2 maps + TOF)		
	**IV-V**	**VI**	**total**

IV	2	-	2

V	3	1	4

VI	1	6	7

total	6	7	13

## Conclusions

These preliminary results show the potential of in-vivo T2 mapping for atherosclerotic plaque characterization. The ability of T2 maps to discriminate plaque components was confirmed by histology.

## Funding

This study was supported by EPSRC and Oxford Biomedical Research Centre.

